# A new simple six-step model to promote recruitment to RCTs was developed and successfully implemented

**DOI:** 10.1016/j.jclinepi.2016.02.002

**Published:** 2016-08

**Authors:** Alba Realpe, Ann Adams, Peter Wall, Damian Griffin, Jenny L. Donovan

**Affiliations:** a Warwick Medical School, University of Warwick, Coventry CV4 7AL, United Kingdom; b School of Social and Community Medicine, University of Bristol, Canynge Hall, 39 Whatley Road, Bristol BS8 2PS, Bristol, United Kingdom

**Keywords:** Recruitment to randomized controlled trials, Orthopedics, Hip impingement, Femoroacetabular impingement, Consultation, Qualitative research

## Abstract

**Objectives:**

How a randomized controlled trial (RCT) is explained to patients is a key determinant of recruitment to that trial. This study developed and implemented a simple six-step model to fully inform patients and to support them in deciding whether to take part or not.

**Study Design and Setting:**

Ninety-two consultations with 60 new patients were recorded and analyzed during a pilot RCT comparing surgical and nonsurgical interventions for hip impingement. Recordings were analyzed using techniques of thematic analysis and focused conversation analysis.

**Results:**

Early findings supported the development of a simple six-step model to provide a framework for good recruitment practice. Model steps are as follows: (1) explain the condition, (2) reassure patients about receiving treatment, (3) establish uncertainty, (4) explain the study purpose, (5) give a balanced view of treatments, and (6) Explain study procedures. There are also two elements throughout the consultation: (1) responding to patients' concerns and (2) showing confidence. The pilot study was successful, with 70% (*n* = 60) of patients approached across nine centers agreeing to take part in the RCT, so that the full-scale trial was funded.

**Conclusion:**

The six-step model provides a promising framework for successful recruitment to RCTs. Further testing of the model is now required.

**What is new?**

**Key findings**
• This study presents a simple six-step model for achieving good recruitment practice, informed by previous research into successful recruitment strategies.
• Despite the challenging nature of the trial, with significant differences in risks and benefits associated with the different treatment arms, a 70% recruitment rate was achieved.

**What this study adds to what was known?**
• While there is an array of established good practice models to guide clinicians in conducting successful diagnostic and treatment consultations in routine clinical practice, the same is not true for trial recruitment consultations.
• The two types of consultations have very different aims and objectives. In routine practice, achieving shared clinician-patient decisions about what treatment is best is of paramount importance, but in recruitment consultations recruiters and patients have to achieve the opposite: equipoise regarding best treatment options, and willingness on the part of patients to become research participants and to receive random treatment allocation.

**What is the implication and what should change now?**
• The six steps are designed to guide how recruitment consultations are conducted.
• The model was developed and used in a pilot trial involving surgical vs. non-surgical treatments, of which few have been conducted.
• Differences between routine and recruitment consultations require an alternative skill set for which recruiters need training and support.

## 1 Introduction

Pragmatic multicentre randomized controlled trials (RCTs) are acknowledged to be the best design for evaluating the effectiveness of health care interventions but often encounter recruitment difficulties [1], [2], [3], [4]. RCTs in surgery face particular challenges, including that many surgeons have limited experience of participating in RCTs, often face learning curves for particular surgical procedures, and sometimes develop individualized rather than standardized techniques. In addition, the comparator for a surgical procedure can often be a very different and more conservative option, such as physiotherapy in orthopedic trials, or no immediate intervention [5], [6].

To participate in an RCT comparing surgery and physiotherapy, all clinicians involved need to accept the possibility that their usual preferred treatment is no more effective than the comparator; and it is particularly difficult for recruiting surgeons to accept this [7]. In addition, discussions about trials are difficult because they may be perceived as disturbing the usual expectations of both patients and clinicians surrounding routine diagnostic and treatment consultations, where shared decisions about best treatment are the intended aim. For patients, the idea that there is uncertainty over the comparative effectiveness of different treatments can also be very difficult to accept [8], [9]. Discussions about trials are therefore awkward and may be avoided, leading to lack of accruals and insufficiently informed patients [7], [10], [11], [12].

Qualitative research methods can be used to understand and inform the development of strategies to improve recruitment to RCTs [13], [14], [15]. For example, the results of an integrated qualitative study led to the rate of randomization of eligible participants rising from 30% to 70% over a 12-month period in the National Institute for Health Research Health Technology Assessment–funded Prostate testing for cancer and Treatment (ProtecT) study [16]. An innovative approach to observing trial information exchange within clinician–patient consultations and giving formative feedback to recruiters based on those observations was among the strategies developed in this study and tested in other RCTs since [10], [13], [17]. Exploring patient preferences, presenting information while being aware of framing effects, and avoiding the use of loaded words were identified as practical actions that recruiters could take to improve recruitment [11], [12], [18], [19], [20].

In this article, we present the development and preliminary testing of a model for trial information delivery that was informed by the findings of the previously mentioned research, within a feasibility study of a trial of arthroscopic surgery for impingement compared with non-operative care study (UK FASHIoN—trial registration: http://www.controlled-trials.com/ISRCTN09754699, [21]). The conservative care arm comprised a detailed physiotherapy protocol developed specifically for the trial, named “Personalised hip therapy” [21]. The aim was to investigate the conduct of recruitment consultations that led to patients agreeing to participate in the pilot, including the order and manner in which the trial information is presented, and comparing this with the content and strategies used in consultations where they did not. Further, we aimed to derive a model from these findings, to offer a simple structure for a recruitment consultation that can be used in RCTs, and also inform the training of clinicians interested in conducting surgical RCTs.

## 2 Methods

The multicentre pilot UK FASHIoN RCT was undertaken in 10 National Health Service hospitals selected because they undertook a high volume of hip arthroscopic surgery. Details of the pilot design and characteristics are presented in Table 1. An integrated qualitative research study was set up to observe recruitment processes with the objective of understanding how any difficulties related to design or conduct could be addressed early, and solutions implemented [16].

### 2.1 Recruitment and data collection procedures

The chief investigator and trial management group (TMG) identified that the consultation where randomization was discussed with patients, the “recruitment consultation,” and the prior consultation when the patient was told their diagnosis of FAI, namely the “diagnostic consultation,” represented pivotal points in recruitment to this trial. Sites were asked to audio record these two consultations for each potential trial participant and were provided with protocols for how to record them and about audio file transfer to the research team. The recordings enabled assessment of the content of information exchanged by recruiters and patients during these two key consultations. A total of 92 consultations relating to 60 individual patients were audio recorded (*n* = 34 diagnostic and *n* = 58 recruitment consultations).

Furthermore, the TMG provided standard training about recruiting patients during site visits. This training included the opportunity for a new recruiter to shadow an experienced recruiter talking about the trial to a potential participant. Advice was given about the importance of addressing patient concerns, presenting the trial arms in a balanced manner, and avoiding the use of words such as “trial” and “randomization,” in accordance to results from previous studies of recruitment to RCTs [11], [13], [16], [19]. Recruiters were provided with crib sheets containing ideas for model answers to frequently asked patient questions about the trial, drawing on what had been learned during prepilot work and best recruitment practice identified by others. As the six-step model gradually emerged from concurrent consultation data analysis throughout the trial, learning about this was ploughed back into recruiter training iteratively.

**Fig. 1:**
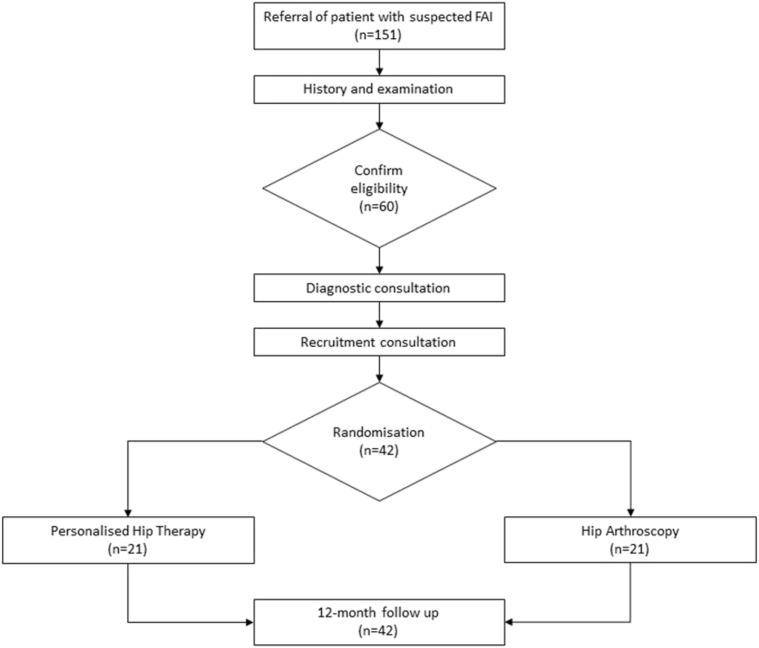
FASHIoN Recruitment Process. *Abbreviation:* FAI, femoroacetabular hip impingement.

Regarding the patient's experience, Fig. 1 shows the recruitment process for FASHIoN. Collaborating surgeons identified potential FAI patients from referral letters. Before their appointment, patients were approached for consent for audio recording of their clinic consultations. Recruiting surgeons assessed patients as usual, taking a history, examining the patient, and performing appropriate imaging investigations. Patients in whom a diagnosis of FAI was made, and who met the RCT eligibility criteria, received a “diagnostic consultation” including a description of the condition from the recruiting surgeon and an explanation of the two possible treatments.

Patients were then provided with a Patient Information Sheet about the pilot RCT, and invited to attend a “recruitment consultation” conducted by a recruiter (e.g., research nurse). Patients had the opportunity to read this document at the beginning of recruitment, engaged in the process of information sharing about the following: FAI and its possible treatments; the pilot RCT and its procedures, including randomization and research follow-up. Patients also had opportunity to ask questions. If considered in equipoise, patients were then invited to give their consent to become participants in the RCT and to be randomized 1:1 to receive either hip arthroscopy or conservative care. The study received ethical approval from the UK National Health Service Research Ethics committee (Ref. No. 11/WM/0389).

### 2.2 Data analysis

Consultation recordings were transcribed and analyzed using the combined techniques of thematic analysis and conversation analysis pioneered in previous studies [20]. The aim was to develop a deeper understanding of recruitment processes, and particularly of communication patterns that were linked to securing patients' informed consent to participate in the trial. The analysis consisted of listening to and reading the transcripts repeatedly to identify and document aspects of information provision that were clear or unclear, sensitive or insensitive to patients' needs, and that either hindered or facilitated recruitment. The content of the consultations was further evaluated to assess whether a logical order and balanced presentation of the RCT arms and other treatment options was given; and whether participants appeared to understand the key issues of equipoise, randomization, participation in the RCT, the option to choose their treatment, and the option to withdraw from the research at any time. Within this framework, common themes emerging from the data were identified, relating to good and bad recruitment consultation practices, and these informed the model's six steps (described in the following paragraphs). Personalized feedback was given to recruiters based on the findings, as in previous studies [16].

Data analysis was carried out prospectively, as consultation recordings became available. The first set of available consultations was analyzed in depth. These comprised audio recordings for 22 individual patients (*n* = 44 consultations), 11 of whom agreed to participate in the trial and 11 did not. The detailed analysis of these patients' recruitment consultations resulted in theoretical saturation (where no new findings emerged for subsequent patient consultations), and so it was decided that further detailed analysis would not be required. The remaining audio recordings were reviewed to check and validate the model described in the next section.

## 3 Results

**Fig. 2:**
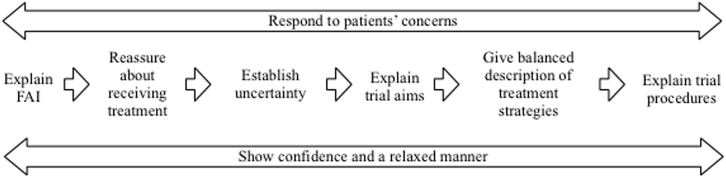
A six-step model for recruitment to an RCT. *Abbreviations:* FAI, femoroacetabular hip impingement; RCT, randomized controlled trial.

The RCT pilot achieved a high proportion of consent, with 60 eligible patients approached, and 42 in 9 sites agreeing to take part in FASHIoN (70%; 95% CI = 58–81%). However, one center did not start recruitment within the time frame of the pilot (i.e., within 18 months). Analysis of the recruitment consultations provided evidence of a logical sequence for information sharing which seemed to facilitate recruitment for both recruiting clinicians and patients. Fig. 2 shows the six-step model that was developed based on the analysis of the audio recordings collected during the FASHIoN pilot trial. It is a guide for recruiters to structure their consultations in such a way as to maximize the likelihood of successful patient recruitment.

The main principle underpinning the model is that recruitment consultations should enable patients to understand the uncertainty arising from a lack of clinical research evidence about the optimal treatment of FAI. This aspect of recruitment consultations is quite different from diagnostic and treatment consultations in routine clinical practice, where the usual aim is for the clinician to remove uncertainty and move toward a shared agreement about the best treatment option. However, in recruitment consultations, there is a need to explain and reiterate that there is uncertainty about the best treatment. Previous research has shown that this is difficult and sometimes uncomfortable for recruiting clinicians [7]. The aim of the six-step model is to provide a framework to facilitate this process. Each step is now explained in more detail, with an emphasis on highlighting what has been learned about best recruitment practice.

### 3.1 Step 1—explain what the condition is to the patient (in this case FAI)

Patients need to receive an explanation about FAI that is easy to understand. We found that successful recruiters tended to use lay terms to explain the morphologic abnormalities related to this condition. They spoke of a “shape abnormality,” an “egg shape,” an “extra little piece of bone,” and so forth. They also made use of metaphors from common everyday experiences when explaining, for example, referring to the piston heads in a car. These attempts to make sure the patient understood what is happening to their bodies were important investments in the relationship and appeared to help patients feel confident in the care they were receiving.

In addition to the “shape abnormality” explanation, successful recruiters also included the role of muscle control in FAI. Explaining this permitted patients to make a logical link between their condition and nonsurgical therapy as a plausible treatment, which could be used later in the discussion. The following quote is an example of a helpful explanation provided by a recruiter:

*Now, lots of people have that sort of shape but only a few people run into trouble with it (…) and my idea on this is if you've got the egg shape, and your muscles are not good at supporting it, then you run into trouble. Now that problem is called femoral acetabular impingement, it's quite a long word, it just means that the ball is rubbing irregularly on the socket. (S01-8)*

Recruiters also introduced to the high incidence of FAI in the population, which supported the sense of urgency about answering the research question and increasing available knowledge about FAI. A direct invitation from the recruiting surgeon to the patient to listen to the recruiter seemed particularly effective in getting the person to consider participation in the trial.

### 3.2 Step 2—reassure the patient that they will receive best treatment

Statements that reassured patients were very powerful in generating trust and openness to joining the trial, for example, when recruiters were confident about having the right diagnosis and explaining their patients would receive the best treatment for their condition. The following quote illustrates the kind of reassurance that patients appeared to value:

*Recruiter: “The ball and the socket are not moving properly together, you're getting a bit of extra rubbing. That rubbing is causing the pain, so there's not really any mystery; we know what the problem is. So, my suggestion is that we treat this problem; I don't think we should just leave it alone, I think we need to try and make you better.” (S11)*

The first two steps of the model, explaining about the diagnosis and that patients will receive the best treatment, set the scene for the discussion about equipoise and the rationale for the RCT.

### 3.3 Step 3—explain that there is uncertainty about which treatment is the best

Uncertainty about which treatment is the best was mentioned early on in the diagnostic appointment and reinforced repeatedly during the consultation. This enabled patients to understand that although there was uncertainty about whether one treatment was better than the other, the two treatments being compared were both effective. This approach helped to reduce patients' uneasiness about not knowing which treatment is the best overall. The following quote is an example of how a recruiter explained this to a patient:

*Recruiter: “Now there are two ways that we can deal with that; one is by surgery, to try and change the shape and the other is by specialised physiotherapy to re-centre the ball in the socket and to control it better. And, I think you know that we are trying to find out which of those is better. We just genuinely don't know which one is better and which one we should recommend to people.” (S53)*

### 3.4 Step 4—explain the purpose of the study

Once uncertainty was established, explaining the purpose of the study followed logically. Recruiters stated how the findings of the research would help clinicians to advise patients like themselves in the future. They also mentioned the need for evidence, emphasizing the real contribution patients could make to advancing science and health care, as illustrated in the following quote:

*Recruiter: “So, here in this hospital, we're running a study to compare the two [treatments] and we're running it at hospitals all around the UK actually, because we recognise that we now know a little bit about this condition, but just not enough to be able to say definitely this treatment's better than the other. So, here we're running the trial to see if we can work out what the best treatment is.”(S34)*.

Successful recruiters were able to harmonize the message that FAI is a condition that clinicians are interested in knowing more about how to treat, simultaneously with the message that patients would be valued, respected, and cared for during the trial, as in the following quote:

*Recruiting surgeon: “My colleague here [indicating recruiter] can explain the study to you, and explain how we try and find out the answer to the question and how you might get involved in that. One thing though, is whatever we do, and I'm going to do everything I can to make you better.” (S10)*

### 3.5 Step 5—give the patient a balanced view about the advantages and disadvantages of each treatment being compared

The trial treatment arms need to be presented in an accessible way, as two methods of dealing with the same condition. Recruiters were asked to spend similar amounts of time explaining each of the arms. Based on the results of the ProtecT trial [20], recruiters were encouraged to start with the nonoperative arm and then to explain the surgical arm. Balance between the presentations of the two treatments was also maintained when talking in detail about the benefits and risks of each arm, for example, the number of visits to hospital required and the duration of the intervention. In the following extract, a good example of a balanced summary of the key aspects of each treatment is presented:

*Recruiter: If it's okay with you, I'll start first of all explaining the nitty-gritty of the personalised hip physiotherapy and then I'll go on and talk about the surgery. [Personalises hip therapy]'s three months in total, it will be different than the physiotherapy that you've had in the past. It's going to be at least six to eight sessions with the senior physiotherapist, who is a specialist in this condition. They're one-to-one sessions. The first [session] will be about an hour long, really doing a good assessment about you and then developing the program from what you tell them and what they identify as being the main areas of concern and what they can improve. Their core exercises are aimed to improve your posture and strength building exercises to help you basically increase the [hip], so moving it slightly up to take the pressure off that area. For the physiotherapy, the risk factors are minimal, at the beginning the pain might get worse before it gets better and that's why they'll work with you to help improve the pain, and they may even recommend you take regular pain killers and anti-inflammatories at the very beginning. It'll involve you having a commitment to do exercises at home.*

*The other option for the study is the surgery, so you've met briefly with [surgeon] who'd be the one who'd be doing the surgery. You'd be asleep for the surgery. You'll be admitted into the hospital overnight. Then they'll have to put it in a little bit of traction and pull your leg. They'll make anything from three to five, little small incisions. They'll either put a little stitch in them and there can be scaring afterwards. You'll be in crutches the next few days to remove the weight from your leg, because that might be painful and to give it time to heal. Most people get off the crutches pretty quickly, some people stay longer than a week, but that's more the exception rather than the rule. Risks involved with any surgery applied, for example there's always the chance that you can get an infection. If you do go ahead and have the surgery, you will separately be consenting to the surgery later on, there'll be another opportunity to speak to the surgeon about particular risks. Quite a lot of people get nerve tingling and numbness after the surgery. For some people it lasts two or three hours, 24 hours, and most people recover from it really quickly. Another risk is that they could break your hip by mistake but that's very rare and it's never happened in this country. Overall it is a very safe operation and most people do fine with it, have a couple of weeks to recover and are back to normal again. (S03)*

Recruiters were encouraged to emphasize the benefits of the two treatments to reassure patients that they were not taking any more risks than if they were making the choice themselves. Where this was done well, patients were usually not sure which treatment to choose. They may have thought about having one treatment at the start of the conversation, but at this point, some expressed either support for the opposite choice or simply uncertainty about which one to choose. Patient equipoise was thus achieved.

In contrast, spending different amounts of time and effort explaining one treatment over the other was associated with consultations in which patients declined to participate in the trial. We observed this framing effect in seven consultations, in which recruiting surgeons introduced hip arthroscopy in greater detail and mentioned physiotherapy and the trial as a somewhat vague afterthought, or not at all.

### 3.6 Step 6—explain the study procedures

Once uncertainty and equipoise were established, the allocation of treatments by randomization seemed easier to explain, and more acceptable. As in previous studies, recruiters were encouraged to explain randomization as a method of allocation to allow a fair comparison to be made [10]. Follow-up questionnaires and patient reassessment after a year were presented as part of a closer follow-up of trial participants than people going through regular care. This was part of the effort made by recruiters to emphasize that patients would be treated with respect and receive personalized care during the research, as illustrated in the following quote:

*Recruiter: “Patients are being so closely monitored. If there are any problems, we can get you back in and we can sort that out.” (S12)*

Two further elements were added to the model, because they were evident at different points throughout consultations resulting in trial participation.

### 3.7 Responding to patients' concerns and questions

Most patient questions occurred after the two treatments were explained. Often patients wanted to know more about the details of each trial arm and the rationale for the nonoperative arm. Successful recruiters usually stopped delivering information to answer questions or concerns raised by patients, before moving onto the next step. This is congruent with a previous study that showed that a highly patient-centered communication style was associated with good patient engagement in clinical trials [11]. Furthermore, failure to respond to patients' concerns or to reassure them that they would receive suitable treatment, as in step 2 of the model, appeared to provoke strong negative reactions toward research participation. The following quote from a patient who refused to take part in the study illustrates this reaction:

*Patient: “I'm not a negative person but the pain gets me quite down and not being able to do things […] if I'd gone to [different hospital] they would just be doing the operation anyway. I feel I've come here and now I'm not quite sure what's going on and to be honest I'm getting very upset” (S05).*

We found stopping to address patient concerns such as these was important for several reasons, including that patients had often waited for a long time to reach the appointment with the recruiting surgeon and needed reassuring that they would receive treatment; as well as affording an opportunity to explain details of the study patients had misunderstood.

### 3.8 Showing confidence and a relaxed manner

Effective recruiters appeared confident and relaxed when talking about the study and were able to make good use of the TMG crib sheets for patients' benefit. The feedback offered to recruiters and tips based on previous research were also important in further encouraging good practice and increasing confidence.

## 4 Discussion

The pilot phase of FASHIoN showed that patient recruitment was feasible and acceptable, although it had been assumed at the outset that it would be extremely difficult (hence the funding body's request for a feasibility study). The qualitative analysis of recruitment consultations highlighted communication practices that led to a high level of patients consenting to participate, with evidence that patients understood the rationale for the trial and its procedures. The six-step model was derived from pilot study findings and informed by previous qualitative research findings [16], [20]. It was further honed during implementation in the FASHIoN pilot trial. Indeed, the feasibility study [21] was considered successful by the funding body, which has now agreed to support the full-scale RCT. The six-step recruitment model will be used to train and support recruiters in the large number of new centers in the full-scale trial. The audio recording of consultations provided evidence to support implementing separate diagnostic and recruitment consultations: the latter being led by research associate or nurse recruiters.

The research presented emphasizes the importance of rethinking the clinician–patient relationship within the context of recruitment to trials. Sharing uncertainty and the equipoise required to participate in this type of research presents challenges to patients and clinicians. Our results add support to the findings of Mills et al. [18], [19] who argue that better information, as well as exploring and gently challenging patient preferences could improve recruitment in RCTs. It also provides further evidence about how the unbalanced presentation of the RCT and the trial arms can hinder recruitment [22], [23]. For recruiting clinicians, difficulties arise when they are not in equipoise, leading them to experience role conflict as recruiters [7]. This conflict was ameliorated in this RCT by the use of the six-step model, which increased patients' confidence in the RCT, including giving reassurance that they would receive effective care in both trial arms.

According to previous research, the most common reasons for patients to decline participating in a surgical RCT are feeling anxious or unhappy about the concept of randomization, preference for a particular treatment option, and receiving previous treatment recommendations from doctors or relatives [10], [19], [20], [24]. The six-step model addresses these common issues by identifying an optimal order for sharing information with patients, which helped them to be more open minded. Randomization, for example, is explained after a thorough explanation of the study purpose and the treatments that are being compared. This arrangement helps patients to understand the reasons behind the study design and, for the majority in the pilot study, it became an acceptable process to determine their treatment. Preferences and previous recommendations were gently challenged during the explanation of both treatments and when addressing patient concerns [18]. Our observation added support to the earlier scientific evidence that the sequence of information presentation can either facilitate or hinder recruitment to clinical trials [20], [23].

Research in health care communication has shown that implementing changes in how clinicians talk to patients is challenging [25], and this is no different within the context of trial recruitment [10]. For example, similarly to our study, Brown et al. [12] developed a stepped approach of ethical strategies that formed the basis of a communication skills training program for doctors in the context of cancer trials. Evaluation of this training showed doctors increased their use of some aspects of shared decision-making behavior and essential ethical information but did not structure their consultations in the recommended FASHIoN [26]. Their approach was successful in modifying some aspects of recruiters' behavior but failed to assist doctors structuring their recruitment consultations. In contrast, preliminary testing has shown that the six-step model is simple to use and provides a helpful framework within which recruiters can organize the presentation of trial information and feel confident in inviting patients to participate. Its use appeared to reduce implementation difficulties reported in many other trials and the stepped approach used by Brown et al. [26] in cancer trials.

Use of the model was supported through provision of specific recruiter feedback and opportunities for new recruiters to watch an experienced recruiter approaching a patient during site visits. Our findings are therefore consistent with previous studies that found that standard and continuing training for recruiters is required to continue to achieve optimal recruitment [13], [26].

Although the six-step model is a way of organizing the presentation of trial information, it is essential that recruiters also take on board the key findings that responding to patients' questions and concerns and developing confidence and a relaxed manner when presenting trial information are vital for recruitment success. These elements of the model provided the right atmosphere, of trust, and opened up opportunities to tackle misunderstandings.

### 4.1 Strengths and limitations

The results of this study should be considered within the context of some limitations. The model was developed based on a relatively small sample of consultations for a specific trial targeting a particular patient group (i.e., young and active working-age adults in the UK). Therefore, it is unknown whether the model can be successfully transferred to other types of RCTs, for example, with higher levels of complexity (e.g., more than two arms) or where prospective participants comprise known “hard-to-reach” groups to recruit from, for example, older, single men [24]. This will be tested in future RCTs, where formal evaluations of the model's effectiveness and cost-effectiveness will be undertaken. However, the six-step model emerged from the pilot study findings and is congruent with findings from a number of other studies [19], [23] and RCTs [20], [22].

We had been concerned that recruiters would be unfamiliar with audio recording their consultations and, even if they agreed to it, might resist making recordings, as others have found previously [17]. However, there was no evidence that this was the case, probably because the audio recordings were introduced at site initiation visits, and adequate information about the recording and transfer of files was developed.

The feasibility study was successful in recruiting participants. Although it is impossible to ascribe cause and effect when so many elements are involved in setting up an RCT, such as site visits, meetings, and so forth, the six-step model did appear to resonate with the pilot study recruiters and to meet their needs. Many had not previously recruited patients to RCTs.

## 5 Conclusion

This study contributes evidence to support the concept that trial recruitment consultations are quite distinct and different, from interactions during diagnostic and treatment consultations in routine clinical practice. We have developed and undertaken preliminary testing of a six-step model of good practice in trial recruitment consultations. The proposed model provides a structured way of delivering trial information and helped the FASHIoN trial to achieve a 70% recruitment rate. The model was helpful for standardizing recruitment practice and enabling patients to give informed consent to participate. The usefulness and completeness of the model will be investigated further during the ensuing main trial and in other trials with recruitment challenges.

## Figures and Tables

**Table 1:** Pilot RCT design and characteristics

RCT acronym	UK FASHIoN
Type	Feasibility
Clinical centers	10
Sample size	60 patient approaches
Interventions	Hip arthroscopy vs. best conservative care
Specialities involved	Surgery and physiotherapy
Primary recruiters	Surgeons, nurses, and research associates
Inclusion criteria	• Age ≥16 • Symptoms of hip pain • Radiographic evidence of femoroacetabular impingement (FAI) on plain radiographs and cross-sectional imaging • Treating surgeon believed they would benefit from hip arthroscopic surgery • Ability to give written informed consent • Ability to participate fully in the interventions
Exclusion criteria	• Previous significant hip pathology • Existing osteoarthritis • Previous FAI surgery

*Abbreviation:* RCT, randomized controlled trial.
